# Neutrophils form extracellular traps in response to *Opisthorchis viverrini* crude antigens, which are elevated in neutrophils from opisthorchiasis patients with hepatobiliary abnormalities

**DOI:** 10.1242/bio.059909

**Published:** 2023-08-07

**Authors:** Krongkarn Watakulsin, Chalida Chuenchom, Chakrit Thapphan, Tran Duong Thai, Sorutsiri Chareonsudjai, Kiatichai Faksri, Sutas Suttiprapa, Sirikachorn Tangkawatana, Banchob Sripa, Steven W. Edwards, Kanin Salao

**Affiliations:** ^1^ Department of Microbiology, Faculty of Medicine, Khon Kaen University, Khon Kaen 40000, Thailand; ^2^ WHO Collaborating Centre for Research and Control of Opisthorchiasis (Southeast Asian Liver Fluke Disease), Tropical Disease Research Center, Faculty of Medicine, Khon Kaen University, Khon Kaen 40000, Thailand; ^3^ Research and Diagnostic Center for Emerging Infectious Diseases (RCEID), Khon Kaen University, Khon Kaen 40000, Thailand; ^4^ Institute of Integrative Biology, Faculty of Health and Life Sciences, University of Liverpool, Liverpool, L69 7ZB, UK

**Keywords:** Neutrophils extracellular traps, *Opisthorchis viverrini*, Neutrophils, Hepatobiliary abnormality

## Abstract

*Opisthorchis viverrini* (*Ov*) infection can cause several disease conditions of the bile duct including hepatobiliary abnormalities (HBAs) and the most severe, cholangiocarcinoma (CCA). Fibrosis occurs when tissues are damaged and normal wound-healing responses are dysregulated. Neutrophils are the first cells to migrate to an infection site to protect the host from intruding extracellular pathogens through a wide range of effector mechanisms such as phagocytosis, production of reactive oxygen species, proteases, or release of neutrophil extracellular traps (NETs). In this work, we used confocal microscopy to assess whether *Ov* crude antigens can cause release of NETs from neutrophils from *Ov*-free individuals. We demonstrated for the first time that these antigens could induce release of NETs *ex vivo* in a dose-dependent manner from neutrophils isolated from *Ov*-free individuals. Intriguingly, when we measured NETs from neutrophils isolated from *Ov*-infected patients, we found increased spontaneous production of NETs in patients with HBAs. Interestingly, exposure to *Ov* crude antigens lowered the level of NETs released by neutrophils from patients with active *Ov* infection regardless of HBA status. We propose that in the case of acute *Ov* infection, even when concentration of *Ov* antigens is relatively low, neutrophils can form NETs. However, when this infection becomes chronic, manifesting as a definite HBA, the levels of NET production are reduced when treated with *Ov* crude antigens. Excessive production of proinflammatory mediators from these NETs might have effects on the parasites, but may also lead to excessive injury of surrounding tissues resulting in HBAs and may lead eventually to the most severe complications such as CCA.

## Teaser

**Summary:** This paper provides novel findings regarding a formation of neutrophil extracellular traps (NETs) in response to liver fluke *Opisthorchis viverrini* crude antigens exposure. We found for the first time that neutrophil extracellular traps can be formed in neutrophils from parasite free individuals, and the level of these traps are associated with the hepatobiliary abnormalities in opisthorchiasis patients.

## INTRODUCTION

Infection with *Opisthorchis viverrini* (*Ov*) or liver fluke is endemic in the Lower Mekong regions of Southeast Asia, including Thailand, with approximately 8 million people infected, especially in the northeast of Thailand where consumption of raw freshwater fishes is common (Sripa et al., 2011). The life cycle of *Ov* requires three different hosts (Smout et al., 2011). First an aquatic snail (first intermediate host) ingests *Ov* eggs from contaminated feces. Asexual reproduction of the parasite produces numerous cercariae, which escape from the snail and penetrate freshwater fish (second intermediate host) (Smout et al., 2011). The cercariae then encyst as metacercariae that are infective to the final definitive hosts including humans. The metacercariae excyst in the duodenum and migrate into the intrahepatic bile ducts to mature (Smout et al., 2011). Infection can cause several disease conditions of the bile duct including cholangitis, cholelithiasis, hepatobiliary abnormalities (HBAs) and the most severe complication, cholangiocarcinoma (CCA) (Elkins et al., 1996; Mairiang et al., 2006, 1992; Mairiang and Mairiang, 2003; Sripa et al., 2007). HBAs occur when tissues are damaged and normal wound-healing responses are dysregulated (Wynn and Ramalingam, 2012), usually as a result of repetitive tissue injury (Borthwick et al., 2013) such as may be caused by *Ov* infection (Mairiang and Mairiang, 2003). To date, it is proposed that that tissue damage caused by *Ov* can be due to either physical damage, release of reactive oxygen species (ROS) from neutrophils, or inflammation (Sripa et al., 2012). However, evidence to support the last of these is scarce or even contradictory.

Neutrophils are the first cells to migrate to an infection site to protect the host from intruding extracellular pathogens. Our group recently reported that the functions of neutrophils were enhanced in patients infected with the liver fluke (*Ov*) and that this increased function was associated with HBAs (Salao et al., 2020), suggesting a double-edged sword role of neutrophils in the liver fluke. Neutrophils act via a wide range of effector mechanisms such as phagocytosis, production of reactive oxygen species, and release of proteases and of neutrophil extracellular traps (NETs) (Nathan, 2006). It was initially thought that NETs are responsible mostly for attacking bacterial infections, especially those in which bacterial biofilms are formed (Khamwong et al., 2022; Thanabalasuriar et al., 2019). Their role in helminth infections is poorly known. NETs consist of decondensed chromatin released from neutrophils together with granular proteins and histones. NETs can be classified into two types namely suicidal and vital NETs. Conventional suicidal NETs are formed after cell death (Fuchs et al., 2007), while vital NETs are formed while neutrophils are still alive (Fuchs et al., 2007). Compelling evidence demonstrates that NETs are released in response to various parasites and cause pathogenesis such as in malaria (Babatunde and Adenuga, 2022; Knackstedt et al., 2019). However, there has been no study of NETs in *Ov* infection with associated HBAs.

In this work, we used confocal microscopy to assess whether *Ov* crude antigens can cause NET release from neutrophils from *Ov*-free individuals. We then asked whether NETs are incorporated with the granule proteins myeloperoxidase (MPO) and neutrophil elastase (NE). In addition, we measured NET release from human neutrophils after challenge with *Ov* crude antigens *ex vivo* from *Ov*-infected patients with or without HBAs.

## RESULTS

### Participant characteristics

Fifty-one Ov^+^HBA^−^ and 22 Ov^+^HBA^+^ patients in this study were from Kalasin Province (Table 1). Most of Ov^+^HBA^−^ patients were female (60.78%), while most patients with HBAs were male (81.82%). The average age of each group was comparable (51.25±6.23 versus 51.14±6.20, *P* =0.680 (Table 1). Levels of *Ov* infection, as measured by eggs-per-gram (EPG) were also comparable between the two groups (18.45±20.53 versus 16.69±14.85, *P* =0.634). All subjects were under 60 years old.

**Table 1.**
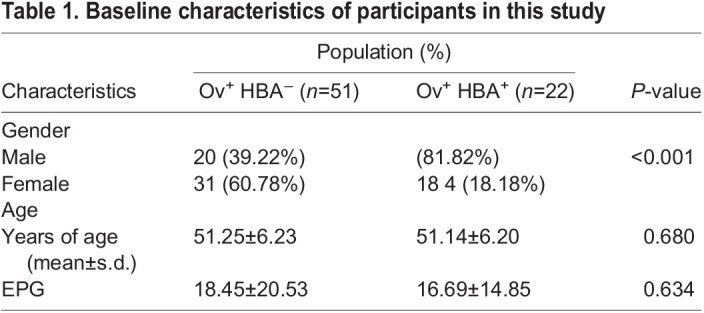
Baseline characteristics of participants in this study

### O*v* crude antigens induced NET production in neutrophils from *Ov*-free individuals without HBAs

**Fig. 1.**
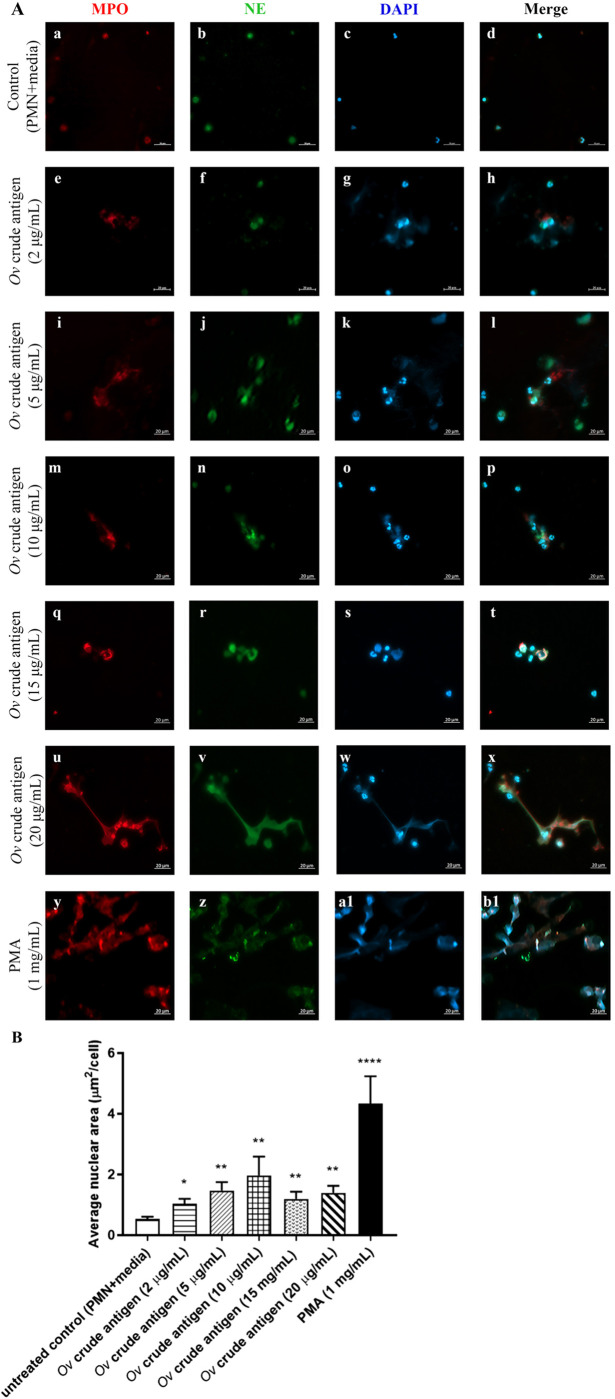
***Ov* crude antigen induced NET production in neutrophils from *Ov*-free individuals.** 1×10^5^ of neutrophils from *Ov*-free and non-HBA individual were confronted with *Ov* crude antigens at final protein concentrations of 2 (e-h), 5 (i-l), 10 (m-p), 15 (q-t) and 20 (u-x) μg/ml and PMA concentration 1 mg/ml (z-b1) for 3 h at 37°C and 5% CO_2_. For confocal microscopy, the cells were stained with MPO (red), NE (green) and DAPI (blue) (representative image from 3 *Ov*-free individual (A). Quantification of NETs was performed after incubation of untreated controls and *Ov* crude antigens (*n*=3) (B). All experiments were performed in duplicate. Area of NETs was measured using ImageJ software. Bars represent mean±s.e.m. All data were analyzed by *t*-test; *P<0.05, **P<0.001 and ****P<0.0001. Scale bars: 20 μm.

**Fig. 2.**
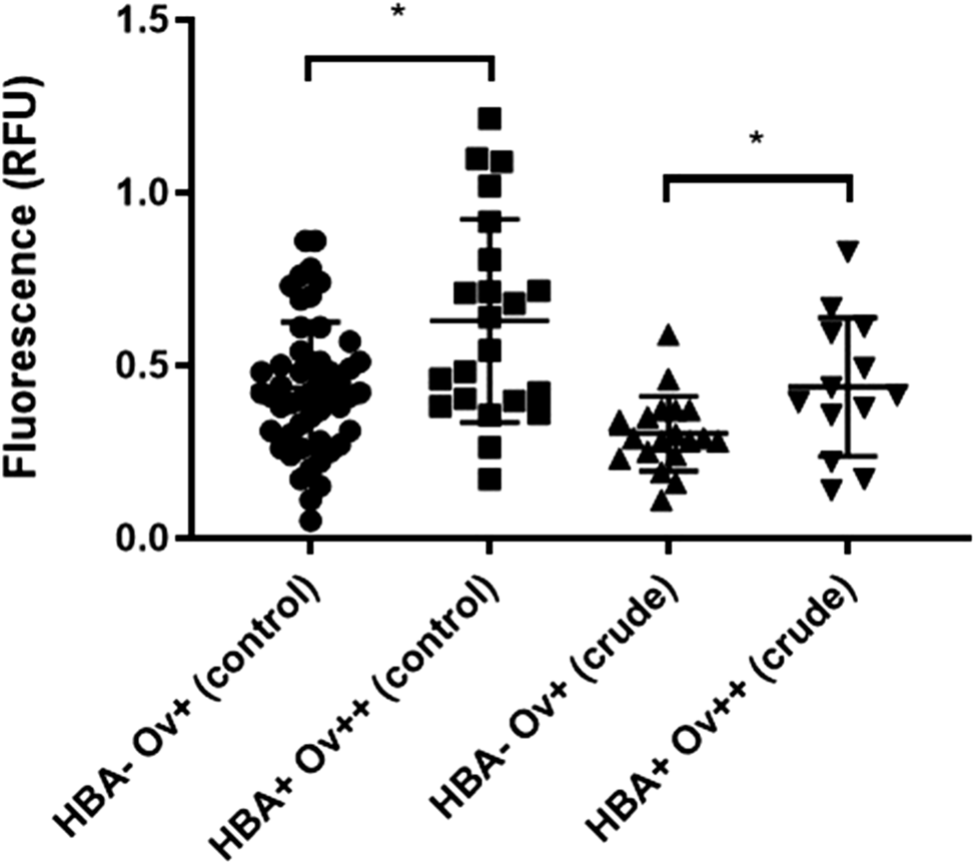
**NETs were elevated in *Ov*-infected patients with hepatobiliary abnormalities.** Neutrophils from *Ov*-infected patients with or without HBA were treated with *Ov* crude antigens (10 μg/ml) and NETs were measured using spectral cytometry. Data are analyzed as mean±s.e.m.; *P<0.05 using *t*-tests (*n*=51 for Ov^+^HBA^−^, *n*=22 for Ov^+^HBA^+^).

To study the effects of different doses of *Ov* crude antigens on NET release, we stained isolated neutrophils from *Ov*-free individual with DAPI. Using confocal microscopy, we observed that human neutrophils release NETs (Fig. 1) when treated with any of the *Ov* crude antigen concentrations tested (2 μg/ml protein (Fig. 1g), 5 μg/ml (Fig. 1k), 10 μg/ml (Fig. 1o), 15 μg/ml (Fig. 1s) and 20 μg/ml (Fig. 1w). As a negative control, we used only culture media instead of *Ov* antigens and used it for a cut-off to determine NET release (Fig. 1C). On the other hand, as a positive control, PMA-treated neutrophils released NETs as expected. Of note, the amount of NET released increased in a dose-dependent manner and peaked at 10 μg/ml of *Ov* antigens before falling at higher concentrations of *Ov* antigens (15 μg/ml and 20 μg/ml) (Fig. 2B). However, the release of NETs at all *Ov*-antigen concentrations was higher than in untreated controls.

To test whether neutrophils that released NETs also underwent degranulation, we stained neutrophils with antibodies against MPO and NE and observed spatial distribution under a confocal microscope. We found that neutrophils when treated with *Ov* crude antigens release NET together with MPO and NE in a dose-dependent manner. We found MPO and NETs starting at 5 μg/ml of *Ov* antigen (Fig. 1l), and of NE and NETs starting at 2 μg/ml *Ov* antigen (Fig. 1h). Interestingly, we observed that all three (MPO, NE and NETs) only when neutrophils were treated with *Ov* crude antigen at concentrations of 15 μg/ml and 20 μg/ml.

### NETs are formed in patients with hepatobiliary abnormalities (HBA+) and *Ov* infection

Because NETs are involved with pathology and severity of parasitic diseases such as malaria (Knackstedt et al., 2019), we sought to investigate if this also true for *Ov*-induced HBAs. We compared the quantities of NET released from neutrophils of individuals without HBAs who were positive for *Ov* eggs in feces (Ov^+^HBA^−^) with quantities from the *Ov* egg-positive and HBA-positive group (Ov^+^HBA^+^). We found elevated levels of NETs in the latter group (Fig. 2). Interestingly, when these neutrophils were challenged with *Ov* crude antigens, we observed lower NET release in both groups regardless of their HBA status.

## DISCUSSION

Neutrophils release NETs upon encounter with large pathogens that usually cannot be phagocytosed (Branzk et al., 2014). In the case of extracellular parasites, especially when the infection is chronic and the infectious organism well evolved with the host, release of NETs might be an inappropriate response, causing bystander tissue damage and contributing to immunopathology. In this work, we decided to test whether *Ov* crude antigens can induce NET formation and release in *Ov-*free individuals compared to patients infected with the parasite with or without HBAs. Our study shows for the first time that *Ov* crude antigens can induce NETs *ex vivo* in a dose-dependent manner from neutrophils isolated from three *Ov-*free individuals. Intriguingly, when we measured NETs from neutrophils isolated from *Ov*-infected patients, we found increased spontaneous NET release in patients with HBAs. Interestingly, treatment with *Ov* crude antigens lowered the level of NETs in patients with active *Ov* infection regardless of HBA status.

There is compelling evidence that neutrophils form NETs in response to helminth and protist parasites: *Ostertagia ostertagi* (Mendez et al., 2018), *Haemonchus contortus* (Munoz-Caro et al., 2015), *Neospora caninum* (Villagra-Blanco et al., 2017), *Eimeria bovis* (Behrendt et al., 2010) and recently *Fasciola hepatica* (Peixoto et al., 2021). In line with these studies, our results show that human neutrophils release NETs in a dose-dependent manner after encounter with crude *Ov* antigens. We observed NETs in response to a very low concentration of *Ov* crude antigens, suggesting this response may be possible *in vivo*, where the actual abundance of parasite antigen can be quite low at the start of acute infection. Although it is unclear whether neutrophils are recruited to the site of *Ov* infection in response to antigens released by the fluke, several studies have reported a rapid recruitment of neutrophils to the site of infection by *Strongyloides sterocoralis* (Galioto et al., 2006) and *Heligmosomoides polygyrus* (Anthony et al., 2006). It is possible that such a recruitment may be prompted by tissue injury caused by parasite larvae and also by parasite-derived chemotactic factors.

*Ov* crude antigens have been reported to activate bile-duct epithelial cells via TLR2 (Yongvanit et al., 2012). Given that ligation of TLR2 results in vital NETs, in which neutrophils are still viable and can perform other functions such as secretion of ROS, it is possible that *Ov* crude antigens may stimulate a similar pathway to release NETs. Previously, we showed that enhanced neutrophil functions, including production of ROS, were associated with HBAs (Salao et al., 2020). Thus, NETs observed in this study further confirm our hypothesis on the association of enhanced innate immunity with development of cholangiocarcinoma (Edwards et al., 2018).

NETs cause tissue injury in liver diseases (Hilscher and Shah, 2020) such as alcohol-associated liver diseases and portal hypertension and cancer. NE from NETs is associated with matrix metalloproteinase-9 (MMP-9) for activation of dormant cancer cells (Albrengues et al., 2018). Likewise, several studies report pro-tumorigenic role of NETs in different cancers (Bravo-Fernandez et al., 1985; Boone et al., 2015). Interestingly, we detected spontaneous release of NETs in patients with HBAs in both *Ov* crude antigen-untreated and -treated groups. These results imply that NETs are correlated with HBAs, as a result of wound healing following tissue damage.

This study has some limitations. First, the NETs we observed only came from *ex vivo* experiments that may not represent what happens *in vivo*. Second, although it is possible that neutrophils may directly interact with live *Ov* during the early phase of infection, our study did not investigate such an interaction. Third, our subjects were all from *Ov*-endemic areas. Past, repeated or chronic infection with *Ov* may have interfered and caused bias to our results. A study of the effects of acute and chronic infection on NET release and formation of HBAs would be worthwhile. Other future studies could include how NETs may form *in vivo* and their effect on cancer development.

In conclusion, we investigated whether human neutrophils could form NETs in response to *Ov* crude antigens. We found that NETs were released at all tested concentrations of *Ov* crude antigens in a dose-dependent fashion and these NETs may be harmful to both the parasite and host. We propose that in the case of acute *Ov* infection, when concentration of *Ov* crude antigens is relatively low, neutrophils could form NETs. However, when this infection becomes chronic, manifesting as HBAs, these levels of NETs were reduced when neutrophils were treated with *Ov* crude antigens. Excessive production of proinflammatory mediators from these NETs might have an effect on the parasites but might also lead to excessive injury of surrounding tissues and hence result in HBAs.

## MATERIALS AND METHODS

### Participants

For NET measurement by confocal microscopy, three *Ov*-free individuals without HBAs who were regular blood donors were recruited from Blood Bank in Srinagarind University Hospital. These individuals donated blood from which neutrophils were obtained.

For NET measurement by spectro cytometry, *Ov*-infected patients were from ten villages in Kalasin Province (Thailand). Individuals aged between 20 and 60 years were recruited into this study. They were separated into two groups: *Ov*-positive patients without HBAs (*Ov*^+^HBA^−^) and *Ov*-positive patients with HBAs (*Ov*^+^HBA^+^). Written informed consent was obtained from each participant. This study complied with the standard good clinical practice (GCP) guidelines and was approved by the Ethics Committee of Khon Kaen University, Khon Kaen, Thailand, reference numbers HE591185 and HE480528.

### Sample-size calculation

A statistical power analysis was performed to calculate required sample size. With an alpha =0.05 and power =0.80, the projected sample size for this effect size (G*Power 3.1.9.2 analysis) was approximately 3 per group.

### Ultrasonography

A detailed description of the ultrasonography methods used in this study can be found in previous publications (Mairiang et al., 2012; Sripa et al., 2009). Using a mobile, high-resolution ultrasound (US) machine (GE model LOGIQ Book XP, GE Healthcare, WI, USA), hepatobiliary abnormalities including portal-vein radical echoes, echoes in liver parenchyma, indistinct gallbladder wall, gallbladder size, sludge and suspected CCA, were graded and recorded. Individuals were classified as not having hepatobiliary abnormalities (“HBA-”) if the US grade was 0 or 1, or as having abnormalities (“HBA+”) if the US grade was 2 or 3. Individuals with alcoholic liver disease, which is seen as fatty liver by US examination, were excluded. Individuals with marked hepatic fibrosis not related to *Ov* infection (e.g. cirrhosis due to hepatitis C and B virus) were also excluded from this study. Our assumption was that remaining types of HBAs in *Ov*-infected individuals were due to chronic *Ov* infection.

### Preparation of *Ov* crude antigens

Adult *Ov* worms from experimentally infected hamsters (previously described in Wonkchalee et al., 2012) were washed three times with sterile phosphate-buffered saline (PBS pH 7.2) containing 0.149 M sodium chloride (Thermo Fisher Scientific, NJ, USA), 8.29 mM disodium hydrogen phosphate (Acros Organics, NJ, USA) and 18 mM sodium dihydrogen phosphate monohydrate (Thermo Fisher Scientific) in deionized (DI) water. A 100× Protease Inhibitor Cocktail (Calbiochem, CA, USA) was added, the mixture (including worms) homogenized using an ultrasonic (MISONIC Sonicator 3000, NY, USA) and then centrifuged at 4°C, 15,000 ***g*** for 30 min. The BCA™ Protein Assay Kit (PIERCE, IL, USA) was used to determine the protein yield of *Ov* crude antigens in the supernatant, which was collected and stored at −80°C until used.

### Neutrophil isolation

Blood was collected from all participants in sodium heparin spray coated tubes (catalogue number #367874, Becton Drive, NJ, USA). Whole blood was mixed with HetaSep™ (#07906, Stem Cell Technologies Inc.) at a ratio of 1:5 and was incubated at 37°C for 30 min until the buffy coat interphase formed approximately 50% of total volume. Neutrophils were isolated from the buffy coat by using Ficoll-Hypaque (#25-072-CV, Corning), at a ratio of 1:1 and centrifuged at 500 ***g*** continuously for 30 min. The granulocyte layer in the bottom was carefully removed and added to RPMI 1640 media (#31800105, Gibco) followed by lysis buffer at a ratio 1:9 to remove erythrocytes, then centrifuged at 500×***g*** for 3 min. The supernatant was discarded, and cells were resuspended in media at a final concentration 1×10^6^ cells/ml.

### NET measurement by confocal microscopy

Neutrophils (approximately 2×10^5^ cells/ml) from *Ov*-free donors (approximately 2×10^5^ cells/ml) were seeded onto sterile round coverslips in 24-well plates. *Ov* crude antigens at various final concentrations (2, 5, 10, 15 and 20 μg protein/ml), or a positive control PMA (#P1585, Sigma) at 1 mg/ml, were added to the 24-well plates and incubated at 37°C for 3 h to allow for NET formation. Cells adhering to the coverslips were fixed with 4% paraformaldehyde and kept overnight. Cells were washed with 1X Tris-buffered saline (TBS) three times for 5 min each. Cells were permeabilized using 0.05% Tween 20 in TBS for 1 min then blocked using 2% bovine serum albumin (BSA) for 30 min and washed three times with 1X TBS. Primary antibody, anti-elastase antibody (#AB21590, Abcam), and anti-myeloperoxidase antibody (#AB109116, Abcam), were diluted (1:200) in blocking buffer then incubated for 1 h. Cells were washed three times for 5 min with 1X TBS. Secondary antibody, goat anti-mouse IgG H&L (Alexa Fluor 488, #AB150113, Abcam), and donkey anti-rabbit IgG H&L (Alexa Fluor 647, #AB150075, Abcam) were diluted (1:400) in blocking buffer then incubated for 1 h and washed a further three times for 5 min with 1X TBS. Cells were stained with DAPI (cat# A1001, Biochemica), which was diluted (1:10,000) in TBS for 3 min. Cells were washed twice with 1X TBS and mounted using 70% glycerol. Cells were then imaged on a confocal laser scanning microscope (Zen 2.1 software, Zeiss LSM800) using 10X and 63X objectives. To quantify the number of NETs, an average nuclear area of neutrophils (μm^2^/cell) was calculated using ImageJ software by the following equation:

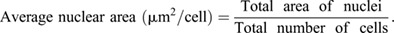


### NET measurement by spectro cytometry

Neutrophils (approximately 2×10^5^ cells/ml) from *Ov*-infected patients were seeded into 96-well plates (Thermo Fisher Scientific). *Ov* crude antigens at various concentrations (10 μg/ml) were added to the 96-well plates and the plates incubated at 37°C for a further 3 h. CaCl_2_ (0.1 M) was then added to stop reactions followed by addition of 50 U micrococcal nuclease (Sigma) and incubation at 37°C for 10 min in order to cleave DNA from the nucleus. The nuclease reaction was stopped by adding 5 μl EDTA (0.5 M). Supernatant containing cleaved DNA was quantified using the QuantiFluor® dsDNA system (Promega, Madison, WI, USA) in black 96-well plates (Thermo Fisher Scientific) using serially diluted lambda DNA as a standard. Measurement was carried out at 485 nm excitation/ 535 nm emission on VarioskanFlash (SkanIt Software 2.4.3 RE for Varioskan Flash).

### Statistical analysis

For data analysis, GraphPad Prism 7 (v. 7.03 h, GraphPad Software, Inc.) and VarioskanFlash (SkanIt Software 2.4.3 RE for Varioskan Flash) were used. The unpaired two-tailed *t*-test was used. All data are presented as mean±s.e.m. Statistical significance was set at *P<0.05; **P<0.01; ***P<0.001; NS, not significant.
